# Inertial-Based Filtration Method for Removal of Microcarriers from Mesenchymal Stem Cell Suspensions

**DOI:** 10.1038/s41598-018-31019-y

**Published:** 2018-08-20

**Authors:** Reza Moloudi, Steve Oh, Chun Yang, Kim Leng Teo, Alan Tin-Lun Lam, Majid Ebrahimi Warkiani, May Win Naing

**Affiliations:** 1 School of Mechanical and Aerospace Engineering, Nanyang Technological University (NTU), 50 Nanyang Avenue, Singapore, 639798 Singapore; 2 Bio-Manufacturing Programme, Singapore Institute of Manufacturing Technology (SIMTech), Agency for Science, Technology and Research (A*STAR), Innovis, Singapore 138634 Singapore; 3 Stem Cell Group, Bioprocessing Technology Institute, Agency for Science, Technology and Research (A*STAR), Centros, Singapore 138668 Singapore; 4 School of Biomedical Engineering, Center for Health Technologies, University of Technology Sydney, Sydney, Ultimo NSW 2007 Australia; 5 Institute of Molecular Medicine, Sechenov First Moscow State University, Moscow, 119991 Russia

## Abstract

Rapidly evolving cell-based therapies towards clinical trials demand alternative approaches for efficient expansion of adherent cell types such as human mesenchymal stem cells (hMSCs). Using microcarriers (100–300 µm) in a stirred tank bioreactor offers considerably enhanced surface to volume ratio of culture environment. However, downstream purification of the harvested cell product needs to be addressed carefully due to distinctive features and fragility of these cell products. This work demonstrates a novel alternative approach which utilizes inertial focusing to separate microcarriers (MCs) from the final cell suspension. First, we systematically investigated MC focusing dynamics inside scaled-up curved channels with trapezoidal and rectangular cross-sections. A trapezoidal spiral channel with ultra-low-slope (Tan(α) = 0.0375) was found to contribute to strong MC focusing (~300 < Re < ~400) while managing high MC volume fractions up to ~1.68%. Accordingly, the high-throughput trapezoidal spiral channel successfully separated MCs from hMSC suspension with total cell yield~94% (after two passes) at a high volumetric flow rate of ~30 mL/min (Re~326.5).

## Introduction

Off-the-shelf (allogeneic) therapies transplanting human mesenchymal stem cells (hMSCs), derived mainly from bone-marrow, adipose tissue, and umbilical cord blood tissue^1^, are widely adopted due to hMSCs’ regenerative, immunosuppressive, and multipotent features^2,3^. The clinical demand for hMSCs is rising significantly, with more than 400 registered clinical trials^4,5^, and the required doses per patient can reach up to 10^9^ cells^1,6,7^. For instance, the number of cells is estimated to be ~10^12^ cells per lot for diseases that need high doses of ~10^8^-10^9^ cells to be delivered. Using multilayer tissue culture flasks cannot meet the demand efficiently for cell therapy products beyond the scale of 100 billion cells^1,8,9^. Thus, embracing alternative methods for *in vitro* cell expansion is necessary. Bioreactors, for scaling up the cultures in 3D rather than scaling out the cell culture flask in 2D, are used as an efficient and cost-effective approach to commercialization^10–12^. Among different adherent cell bioreactors, employing suspension scaffolds so-called microcarriers (MCs), ~100–300 µm in diameter, within a stirred tank has been widely recognized^7,13^; recently it was demonstrated within a 50-L bioreactor that a 43-fold expansion of hMSCs could be reached in 11 days^14^. Using microcarriers, however, necessitates clarification of cell suspension bulk and downstream removal of MCs.

Following cell expansion and detachment from microcarriers, existing systems for separation of MCs and cells are tangential flow filtrations (TFF), counter-flow centrifugation elutriations (CCE), and dead-end sieving^8^. However, clogging (cake formation) and high shear stress for sieve-based systems^15,16^, as well as high operative costs due to bulkiness and rotating parts for CEE systems such as KSep platform (Sartorious), pose disadvantages. Herein, we report on the advancement of an alternative method using inertial focusing – shown recently to be scalable for filtration of large-scale lot size in the order of liter per min^17–20^.

The inertial focusing phenomenon is only reliant on hydrodynamic forces, therefore, it gives rise to the relatively ease of parallelization to scale out the throughput. A high-throughput cell retention device was recently introduced; it utilized spiral channels for perfusion bioreactors while the projected device footprint for overall ~1000 L perfusion rate during one day was approximated to be 100 mm × 80 mm × 300 mm^17,18^, noticeably smaller when compared to other CEE systems. Furthermore, the inertial-based filtration is a continuous clog-free (or membrane-less) system thereby sustaining reliable steady performance without declining during long-term operation, and obviating the need for filter replacement. In this work, we first systematically investigated inertial focusing of microcarriers in scaled-up spiral channels (channel size ≥ 0.5 mm). Afterward, removal of microcarriers from hMSCs suspension was accomplished by inertial focusing with ~99% purity while cell harvest yield reached ~94%.

## Design Principle

**Figure 1:**
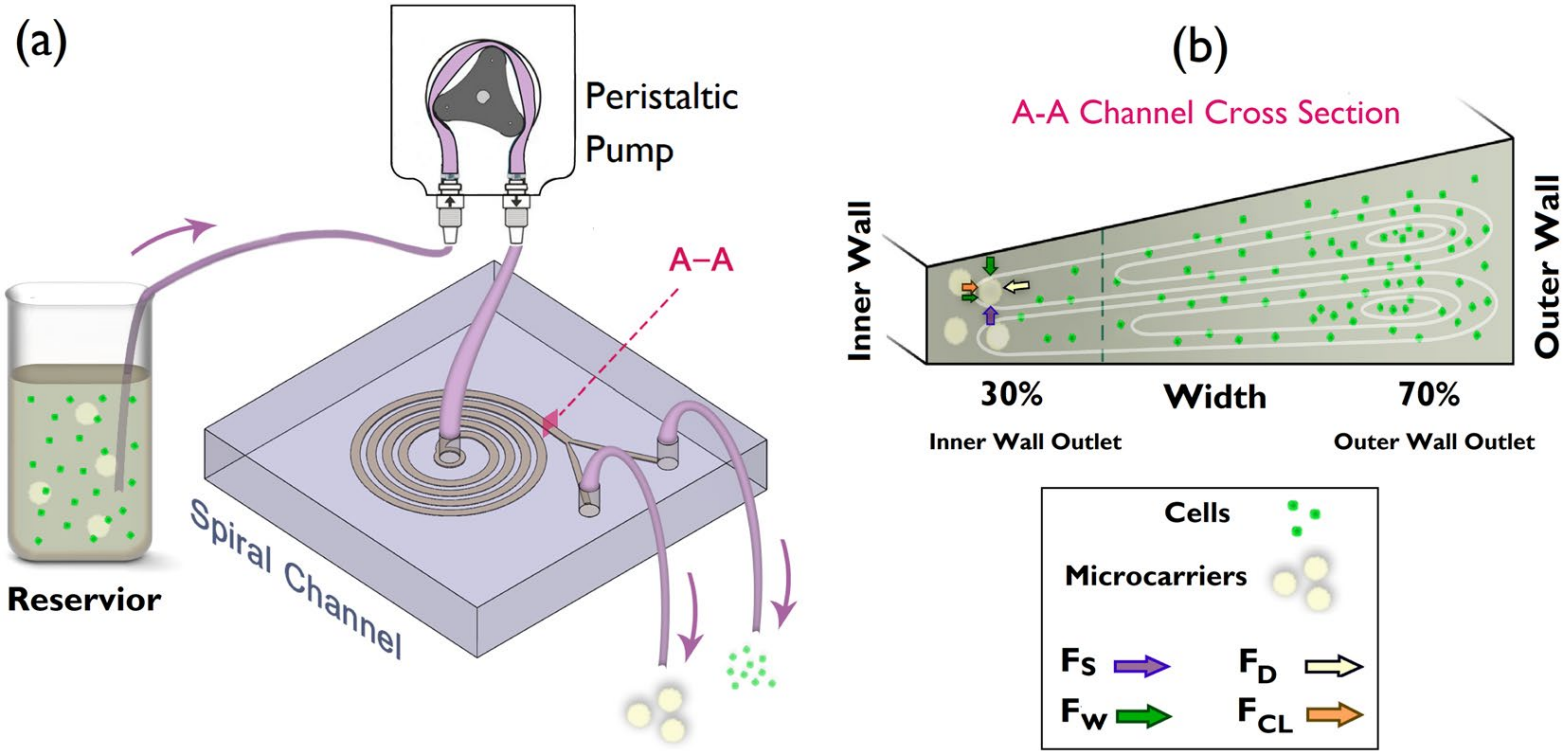
Schematic model showing filtration of microcarriers from cell suspensions. (**a**) The microcarrier-cell suspension is driven to the channel using a peristaltic pump. (**b**) Channel cross-section A-A before bifurcation. Microcarriers equilibrate near the inner wall (IW) by the balancing forces shown schematically whereas cells are mainly circulated by secondary flow and harvested through the outer wall (OW) outlet.

Inertial focusing for neutrally-buoyant particles flowing inside a channel occurs when the particle radius is comparable to the channel hydraulic diameter, *a*/D_H_ > 0.07^21^, and the channel Re number is of order about ~100^22,23^, thereby triggering the shear-gradient lift force (F_S_). Adding any curvature to the straight channel such as a spiral leads to the development of two counter-rotating vortices whose structures rely upon the Re number and channel cross-sectional shape^24–27^. The interplay between the induced shear-gradient lift forces and the secondary drag forces (F_D_) results in distinctive lateral focusing points inside the spiral channels, which is strongly dependent on particle size^28,29^. When the particle approaches the channel walls, wall-induced lift force (F_W_) and cross-lateral wall-effect lift force (F_CL_) emerge^30^. Figure 1b illustrates schematically the balancing forces that develop equilibrium near the inner wall. Since the difference in size between MCs (~100–300 µm) and hMSCs (~10–30 µm) is significant (Fig. 1a), it is impossible to focus both particle sizes simultaneously in a channel. A negative selection approach, therefore, is chosen to focus and filter out the MCs. However, the cells are not large enough to undergo shear-gradient lift forces (*a*/D_H_ ≈ 0.02); as a result, they are mainly influenced by the secondary flow and disperse inside the spiral channel (Fig. 1b). Consequently, some cells are elutriated through the inner wall (IW) outlet. Thus, to enhance cell harvest yield, recirculating IW outlet collection containing both MCs and cells is mandatory. However, because cells are the final product, their viability and integrity are prone to decline when pumped through the channel, as they are iteratively exposed to shear stress. The number of passes to retrieve cells from MCs (IW) outlet, therefore, should be limited. To achieve cell harvest greater than 90% with two passes, it requires a higher amount of cell harvest via outer wall (OW) outlet than 50% for one pass.

**Figure 2:**
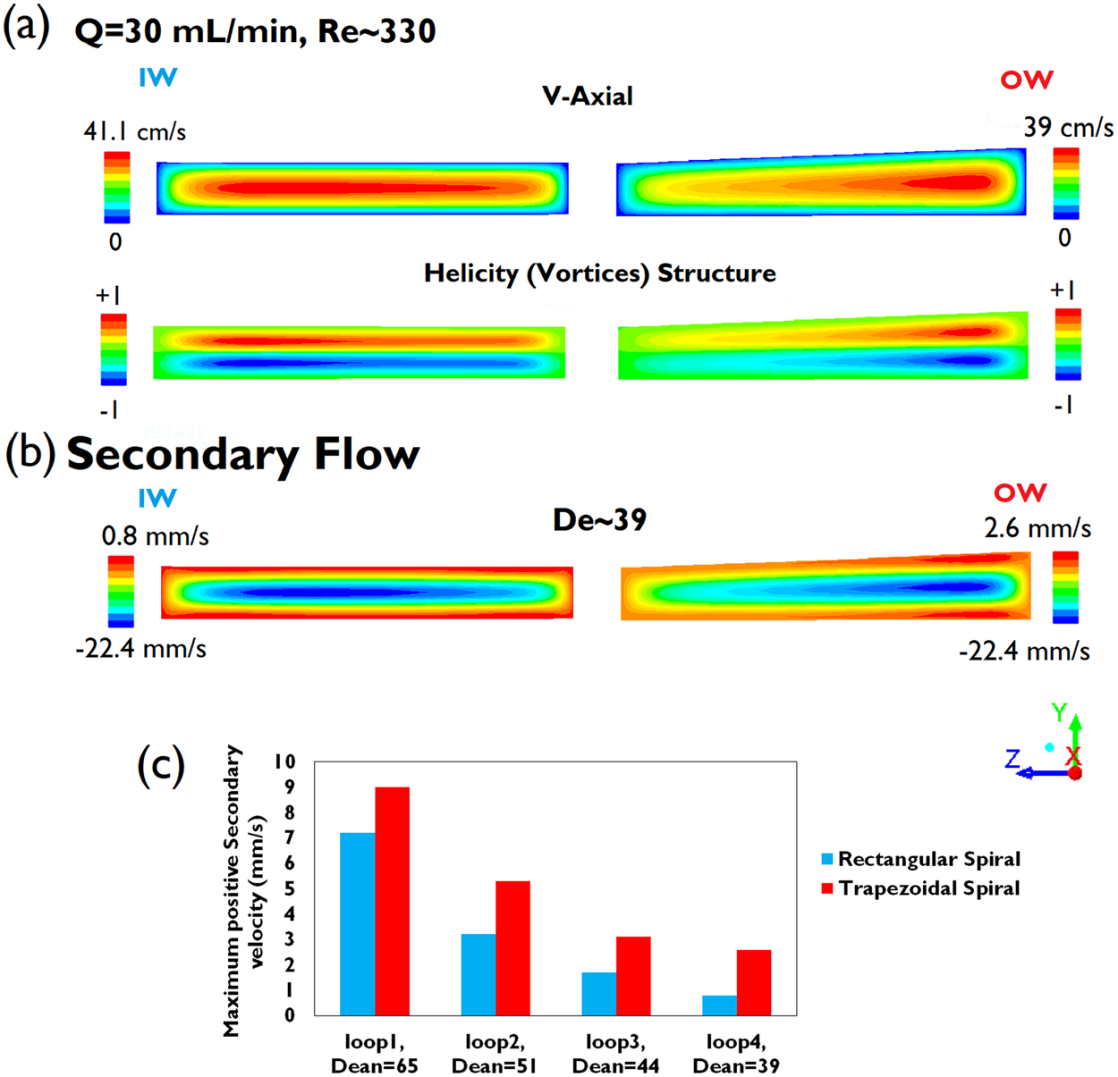
(**a**) Axial velocity profile (+X direction) and vortices structure (Helicity contour) in the rectangular and trapezoidal channels with Tan(α) = 0.0375. (**b**) Secondary flow distribution and (**c**) its maximum positive secondary flow velocities, whose direction from outer wall to inner wall, reduce notably at different loops of the scaled-up rectangular and trapezoidal spiral channel at flow rate of 30 mL/min, but the trapezoidal spiral shows relatively higher positive secondary velocity (+Z direction) and as a result larger drag force to drive particles to the inner wall.

Altering the cross-sectional shape of spiral channels from rectangular to trapezoidal shifts the maximum axial velocity and vortex cores toward the longer side wall (Fig. 2a), thereby elevating the ratio of outer outlet to inner outlet collection from 1:1 up to 2.5:1, depending on the location of bifurcation and channel aspect ratio; it implies the increased harvesting of carrier fluid from outer outlet from 50% up to ~70%. Cell harvesting yield is proportional to elutriated carrier fluid from outer wall outlet assuming uniformly dispersed cells inside the channel although the vortex cores near the outer wall potentially trap and maintain higher cell concentrations locally. Accordingly, trapezoidal spiral channels with different slanted wall slopes are investigated as well as the rectangular spiral for which the slope of the slanted wall is zero (Tan(α) = (H_outer wall_ − H_inner wall_)/W = 0).

To scrutinize secondary flow structure, rectangular and trapezoidal spirals (Tan(α) = 0.0375) with large channel aspect ratios of 8 (AR = W/H) were numerically assessed using ANSYS Fluent to solve the Navier-Stokes equations for incompressible flow (The density, 998.2 kgm^−3^, and dynamic viscosity, 0.001002 kgm^−1^s^−1^, were set for liquid water at room temperature 20 °C). The results show that the trapezoidal spiral not only enhances a positive secondary flow, which contributes to driving particles (or MCs) to the inner wall, but also alters the distribution of secondary flow. As such, the maximum secondary flow’s regions are laid at the outer half of channel cross-section (Fig. 2b). Compared to the corresponding rectangular spiral, the positive secondary flow tapers at a slower rate across the trapezoidal spiral channel, due to the rapid reduction of Dean Number ($De=Re\sqrt{{D}_{H}/2R\,}$ where Re is channel Reynolds number, D_H_ and R are channel hydraulic diameter and radius of curvature respectively) by 60% across the spiral channels. In other words, the difference in positive secondary flow between two spirals increases particularly at the downstream loops (3^rd^ to 4^th^ loop), as shown in Fig. 2c. This illustrates the enhanced secondary flow drag (F_D_~U_D_ where U_D_ is secondary velocity) sweeping particles (microcarriers) toward the inner wall to establish focusing only in an ultra-low-slope trapezoidal spiral (Results Section). Because inertial focusing of MCs near the inner wall cannot be interpreted solely as a result of positive secondary flow without considering the shear force; we investigated MC focusing dynamics experimentally due to the lack of a shear-gradient force model exclusively for spiral channels.

## Material and Methods

### Channel fabrication

Aluminum master molds were fabricated via micro-milling technique (Whits Technologies, Singapore). After casting the mixed polydimethylsiloxane polymer (PDMS, Sylgard 184 Silicone Elastomer Kit, Dow Corning) and curing agent (10:1 ratio) into the mold, it was cured for 30 min in an oven with 80 °C. To boost bonding, we used semi-cured PDMS bonding rather than the conventional oxygen plasma treatment ^31,32^. Following peeling off the semi-cured PDMS spiral channel and punching the inlet and outlets, the PDMS spiral chip was bonded irreversibly with another semi-cured slab of PDMS (~3 mm thickness cast in a petri dish). Subsequently, the assembled channel was further cured for 3 hours in the oven until complete bonding was achieved.

### Cell culture

Bone-marrow-derived hMSCs (Lonza, Singapore) were cultured in T175 tissue culture flasks pre-coated with 0.1% (1 g/L) type A gelatin (Sigma-Aldrich, Singapore) in a humidified incubator with 37 °C and 5% CO_2_ in air. The BM-hMSCs were passaged in high-glucose Dulbecco’s modified Eagle’s medium (DMEM, Life Technologies Holdings Pte Ltd, Singapore) supplemented with 10% fetal bovine serum (FBS, Life Technologies Holdings Pte Ltd., Singapore), 1% Penicillin-Streptomycin (10,000 U/mL, Life Technologies Holdings Pte Ltd) and 10 ng/mL bFGF (Peprotech, Singapore). At ~80% confluency, cells were washed with Ca^2+^- and Mg^2+^-free DPBS, then dissociated using 0.25% trypsin and 0.53 mM EDTA, and finally incubated for 3 minutes. The detached cells were subsequently resuspended in the culture medium (~5-fold dilution) to neutralize the trypsin, then centrifuged with 300 g for 10 min. The harvested cell pellet was then resuspended in the culture medium based on desired cell concentration. The hMSCs were seeded at density of 5000 cells/cm^2^.

### Microcarrier and sample preparation

Dry Cytodex 3 microcarriers (GE Healthcare) were hydrated using 1X Ca^2+^- and Mg^2+^-free phosphate buffered saline (PBS) for 3 hours. Afterward, they were washed twice using PBS prior to autoclaving. The stock solution was prepared at a concentration of 10 g/L. According to the manufacturer’s instruction, the average number of MCs is approximately 3 × 10^6^ MCs/g, which corresponds to 8.42% v/v solid volume fraction (the average diameter is ~175 µm). The stock MC suspension was prepared with various dilution factors to acquire lower volume fractions which are 0.2%, 0.4%, 0.8% and 1.68%.

### Device characterization

**Figure 3:**
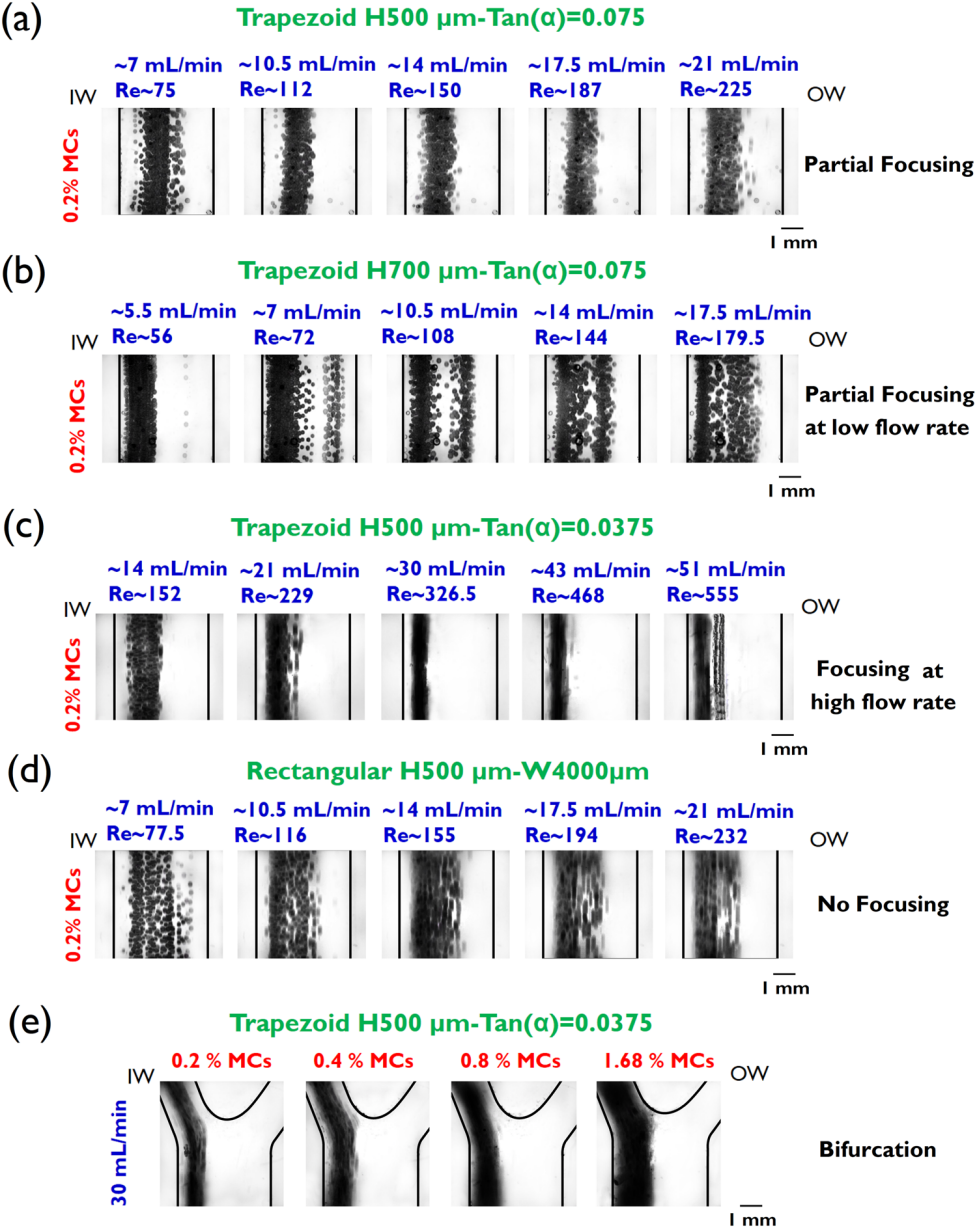
Microcarrier focusing dynamics at the last loop of spiral channel in (**a**) the trapezoid with H = 500 µm Tan(α) = 0.075, (**b**) H = 700 µm and Tan(α) = 0.075, (c) H = 500 µm Tan(α) = 0.0375 and (**d**) H = 500 µm, rectangular cross-section (Tan(α) = 0.0). (**e**) Filtration of microcarriers from the inner wall outlet at 30 mL/min using trapezoidal spiral H = 500 µm Tan(α) = 0.0375. It demonstrates complete removal of microcarriers for high microcarrier volume fraction of 1.68%, while collecting 70% of carrier fluid from the outer wall outlet. Focusing and partial focusing refer to particle band width (BW) <~4*a* and ~4*a* < BW < ~10*a*, respectively.

First, to evaluate focusing dynamics of MCs in spiral channels without cells, the desired MC suspension was dyed with trypan blue (0.1% v/v) to enhance contrast for bright field microscopy. The spiral channel was mounted on an inverted epifluorescence microscope (Olympus IX71, Olympus Inc., USA) which is equipped with a 16-bit CMOS camera (optiMOS, QImaging), and the MC solution was introduced to the channel inlet by means of a peristaltic pump (LeadFluid, BT300S). The MC solution was agitated to prevent deposition. The exposure time was set as low as 100 ms. Image sequences (an average of 200) were stacked to develop a composite image using ImageJ software to obtain the entire footprint of the MC band width (please see video S1–3 that show stacked images for the rectangular and trapezoidal spiral with Tan(α) = 0.0375 in Fig. 3 for 0.2% MCs).

The best spiral design achieved with regards to MC focusing was used for removal of MCs from cell suspensions. To reduce contaminations, the filtration process was carried out inside a biosafety cabinet (BSC). The channel and tubing were sterilized running 70% ethanol for ~15 min. Next, the channel was washed with Ca/Mg-free DPBS for ~10 min to wipe residual ethanol and to keep the channel and tubing filled. Finally, the inlet tubing was inserted into the MC-cell solution previously prepared, and the filtration began by pumping the solution until the total volume reached 45 mL (Fig. S1 shows the experiment setup). Lastly, to enhance cell harvest yield, collected samples from the inner wall (MCs) outlet were once more passed through the channel.

### Cell characterization

#### Cell viability and proliferation

Following cultivation of sorted cells at day 1 in a 6-well tissue culture plate at a concentration of 5 × 10^4^ cells/well, the viability and proliferation of attached cells were examined by PrestoBlue assay. A mixture of 10X PrestoBlue reagent (Invitrogen, Life Technologies Corporation) and culture medium with ratio of 1:10 was prepared. Having been washed in PBS with Ca^+2^/Mg^+2^, each well was filled up with 2 mL of the 1X PrestoBlue reagent stock prepared. Next, the 6-well plate was covered in aluminum foil (to protect from light) and incubated for 40–60 min. The supernatant was then aliquoted (at least 4 samples from each well) to a 96-well plate (100 µL) so to read fluorescence in a plate reader (Tecan Infinite M1000). Finally, the PrestoBlue reagent was washed off in PBS without Ca^+2^/Mg^+2^, and the 6-well plate was topped up with fresh culture medium and placed in the incubator.

#### Trilineage differentiation

Once the sorted cells had grown to 80% confluency, they were harvested using the methods described in Cell culture.

Adipogenic differentiation: Prior to cell seeding for adipogenic differentiation, a 24-well plate (Nunclon Delta Surface, Thermo Scientific) was pre-coated with fibronectin (Sigma-Aldrich). 200 µL of Ca^2+^- and Mg^2+^-free DPBS, and 2 µL of fibronectin were added to each well. The plate was incubated at 37 °C for 1 hour, after which the coating solution was aspirated. 6 × 10^4^ cells in 500 µL of culture medium (described in Cell culture) were seeded into each well. After 24-hour incubation, all the culture medium was aspirated and the wells rinsed with Ca^2+^- and Mg^2+^-free DPBS twice. 500 µL of adipogenic differentiation media (MSCgo^TM^ Adipogenic, Biological Industries) was added to the wells. The plate was incubated at 37 °C for 28 days, with 100% medium change done every 2 or 3 days.

Oil Red O staining for adipogenic differentiation: At the end of 28 days of culture, the adipogenic differentiation medium was removed from the 24-well plate and the wells were rinsed with Ca^2+^- and Mg^2+^-free DPBS once and removed. 500 µL of 4% formaldehyde (Paraformaldehyde Solution, 4% in PBS, Affymetrix/USB) was added to each well; the plate was then incubated at room temperature for 1 hour. 0.175 g of Oil Red O (Sigma-Aldrich) was dissolved in 50 mL of 2-propanol (Sigma-Aldrich) and filtered through a 0.2 µm filter (Minisart®, Sartorius Stedim Biotech). 18 mL of the dissolved Oil Red O was mixed with 12 mL of deionized water and was left at room temperature for 20 minutes. The staining solution was filtered through a 0.2 µm filter. 4% formaldehyde was removed from the fixed samples and samples were rinsed with Ca^2+^- and Mg^2+^-free DPBS. 1 mL of Oil Red O staining solution was added to each well. The plate was incubated at room temperature for 30 minutes, after which the stain was removed, and the wells were rinsed with deionized water until excess Oil Red O stain was washed off. The plate was imaged at 4X, 10X, and 20X magnification (EVOS FLc, Life Technologies).

Chondrogenic differentiation: 1 × 10^5^ cells in 100 µL of culture medium (described in Cell culture) were seeded into each well of a 96-well ultra-low attachment, round-bottom plate (Ultra Low Cluster, 96 Well, With Lid, Round Bottom, Ultra Low Attachment Polystyrene, Costar) and incubated for 24 hours. Culture medium was subsequently aspirated and rinsed with Ca^2+^- and Mg^2+^-free DPBS twice. 200 µL of chondrogenic differentiation media (MSCgo^TM^ Chondrogenic XF, Biological Industries) was added to the wells. The plate was incubated at 37 °C for 28 days, with 100% medium change every 2 or 3 days.

Alcian Blue staining for chondrogenic differentiation: After culturing for 28 days, chondrogenic differentiation medium was removed from the 96-well plate, and all wells were rinsed with Ca^2+^- and Mg^2+^-free DPBS once. 200 µL of 4% formaldehyde was added to each well, and the plate was incubated at room temperature for 1 hour. 0.2 g of Alcian Blue 8GX (Sigma-Aldrich) was dissolved in 20 mL of 0.1 N hydrochloric acid (Sigma-Aldrich) and filtered through a 0.2 µm filter. 4% formaldehyde was removed from the fixed samples and samples were twice rinsed with deionized water. 200 µL Alcian Blue staining solution was added to each well. The plate was incubated at room temperature overnight and protected from light. The Alcian Blue stain was removed and the wells were rinsed with 0.1 N hydrochloric acid until excess Alcian Blue stain was washed off. 200 µL deionized water was added to each well. The plate was imaged at 4X magnification.

Osteogenic differentiation: Before cell seeding for osteogenic differentiation, a 24-well plate was coated with fibronectin, as described in adipogenic differentiation. 6 × 10^4^ cells in 500 µL of culture medium were seeded into each well and incubated for 24 hours. After a 24-hour incubation period, all culture medium was aspirated and rinsed with Ca^2+^- and Mg^2+^-free DPBS twice. 500 µL osteogenic differentiation medium (MSCgo^TM^ Osteogenic XF, Biological Industries) was added to the wells. The plate was incubated at 37 °C for 28 days, with 100% medium change every 2 or 3 days.

Alizarin Red S staining for osteogenic differentiation: After 28 days of culture, osteogenic differentiation medium was removed from the 24-well plate, and all wells were rinsed with Ca^2+^- and Mg^2+^-free DPBS. 500 µL cold 70% ethanol was added to each well, and the plate was incubated at room temperature for 1 hour. 1 g Alizarin Red S (Sigma-Aldrich) was dissolved in 50 mL of deionized water. The pH was adjusted to 4.1–4.3 using 0.1 N hydrochloric acid or 0.5 M sodium hydroxide (prepared from sodium hydroxide pellets from 1^st^ BASE) and filtered through a 0.2 µm filter. Ethanol was aspirated from samples and each was rinsed with deionized water thrice. 500 µL Alizarin Red S stain was added to each well, and the plate was incubated at room temperature for 30 minutes. The stain was removed, and the wells were rinsed with deionized water until excess stain was washed off. 500 µL deionized water was added to each well. The plate was imaged at 4X and 10X magnifications.

## Results and Discussion

### Microcarrier focusing dynamics in the scaled-up spirals

A series of scaled-up trapezoidal spiral channels were fabricated to investigate the effects of channel height and slanted wall slope – Tan(α). The cross-sectional shape of fabricated channels can be seen in Fig. S2. The initial radius of curvature measured from the inner wall was 5 mm, and the pitch of the Archimedean spiral was 7 mm (3-mm interval between successive spiral loops). The channel width was fixed at 4 mm while the channel heights varied from 0.5 mm to 1 mm. Higher channel hydraulic diameter through increasing either channel height or width causes non-focusing or partial focusing of microcarriers (data is not shown). The channel Reynolds number was defined based on maximum shear rate: ${R}_{e}={\rho }_{f}{U}_{max}{D}_{H}/\mu \,$(where ρ_*f*_ is the density of fluid, U_max_ ≈ 3/2 U_avg_^21,28^, D_H_ is the hydraulic diameter of channel and µ is the dynamic viscosity of fluid).

Figure 3a–d are the composite bright field images illustrating the complete footprint of MC focusing before bifurcation. First, we investigated a scaled-up trapezoidal spiral with a low slant (Tan(α) = 0.075) that is similar to trapezoidal spirals reported at the micron scale^25,33^. Though the MC volume fraction is as low as ~0.2%, the relatively large band of MC streaks equilibrated at the inner half of the cross-section (Fig. 3a) demonstrates malfunctioning of the channel (0.5 mm inner wall height and 0.8 mm enlarged outer wall).

It should be noted that the channel is fit for low-volume-fraction suspensions while particles are collected from the inner wall outlet (See Fig. S3 displaying the filtration of MCs with low V_f_ ≈ 0.1%). Similar to trapezoidal spiral channels at micron scale when a small population of larger particles/cells is separated from an abundant population of smaller particles/cells–e.g. separation of CTCs from WBCs or WBCs against RBCs population–^33,34^ the channel performs robustly. Conversely, the MC volume fraction is not limited to ~0.1% or less here. In addition, unlike the present configuration where the Dean number ($De=Re\sqrt{{D}_{H}/2R\,}$) declines rapidly by ~60% across the spiral channel, the increasing-Dean mode of the trapezoidal spiral (switching the direction of MC solution pumped) was examined. Though it enhances focusing but it still cannot handle the high MC volume fraction of greater than 0.4% (Fig. S4).

Next, effects of particle clogging ratio were investigated by increasing channel height. Importantly, though there is no theoretical limitation on channel height when the ratio of particle size to channel height (K = a/H) ranges typically from 0.1 to 0.4, a 0.2 mm increase in average channel height led to MCs equilibrating partially at significantly low Re number ~56 (Fig. 3b, 5.5 mL/min). The corresponding average K factor decreased from 0.26 to 0.20. The physical ramification of reduced volumetric flow rate was a lower velocity of particles traveling through the channel (flow velocity U_avg_~2.6 cm/s), which heightened the potential of clogging the channel, particularly when the microcarrier volume fraction was high (V_f_ > 0.4%).

Finally, we investigated variations in the slope of the slanted spiral wall. Remarkably, Fig. 3c demonstrates that a trapezoidal spiral wall with an ultra-low-slope slanted wall (Tan(α) = 0.0375, channel height varied from 0.5 mm to 0.650 mm at outer wall) could only establish the MC focusing (particle band width < 4*a*) close to the inner wall at a noticeably high flow rate of ~30 mL/min (Re~326.5). The MC dispersion began at Re greater than ~468, however, the swift switch in equilibrium position from the inner wall to the outer wall was not observed even at high Re~555 compared to conventional trapezoidal spirals at micron scale^25,33^. The ultra-low slanted wall and relatively large particle clogging ratio could potentially delay the transition of focusing from the inner wall to the outer wall. The corresponding rectangular spiral channel (Fig. 3d, Tan(α) = 0) verified the constructive impact of the ultra-low-slope slanted wall on MC focusing, while even partial focusing was not formed regardless of channel Re number. It should be noted that the elevated channel height in Fig. 3c is relatively small in the order of MC size or even lower (150 µm) particularly at the outer half of channel cross-section in the ultra-low-slope trapezoidal spiral. As previously demonstrated numerically (Fig. 2), in addition to the enhanced secondary flow’s drag force overall, the ultra-low-slope trapezoidal spiral may cause the MCs exposed further to the positive secondary flow close to the top and the bottom wall than the typical trapezoidal spirals with a larger slope (Tan(α)~0.1), i.e. it could alter the equilibrium planes next to the top and the bottom wall relative to the zero-secondary-flow boundary, in which the direction of secondary flow switches, or vice versa.

In conclusion, Fig. 3e demonstrates the performance of the ultra-low-slope trapezoidal spiral in MC filtration efficiency with approximately ~99% (Table S1 Supplementary, efficiency $E=\frac{{C}_{s}-{C}_{o}}{{C}_{s}}\times 100$ where C_S_ is the MC concentration in the reservoir and C_O_ is the MC concentration in the outer wall outlet). The fraction of flow which exited through the outer wall outlet (MC-free outlet) was maintained at ~70% of the carrier fluid that directly impacts on cell harvesting yield. The strong MC focusing developed adjacent to the inner wall, enabled higher MC volume fractions of up to ~1.68% to be removed without compromising filtration efficiency. It is of great importance to incorporate high-volume-fraction MC solutions as industrial scales of MC-based bioreactors (~100 L) have been reported that using MCs with volume fractions in the order of ~1% or more practically^16,35–41^. Thus, the proposed channel streamlines the filtration procedure and eliminates any large dilution factors.

### Removal of microcarriers from mesenchymal stem cell suspension

Since hMSCs are prone to morphology damage and cell death due to high shear stress, it is critical to ascertain viability and cell harvest yield after each pass. A case model, reported recently by Hewitt *et al*.^37^, with 1 g/L of Cytodex 3 (0.84% volume fraction) and ~2 × 10^5^ cells/mL was considered. To enhance cell harvesting, following the first pass, the inner wall (MCs) outlet collection was passed through the ultra-low-slope trapezoidal spiral. To avoid diluting collected sample from MCs outlet after the first pass–the concentration factor is ~3.3 for the IW outlet collection–and reduce delay in harvesting cells from IW outlet for the second pass, ~4X dilution factor was considered. Thus, a 45 mL MCs solution with ~0.22% volume fraction was prepared using cell culture medium. A total viable BM-hMSCs of ~3 × 10^6^ was added to the reservoir containing MCs, a magnetic stirrer was used to gently agitate the MC–cell solution (average cell concentration was ~0.67 × 10^5^ cells/mL).

**Figure 4:**
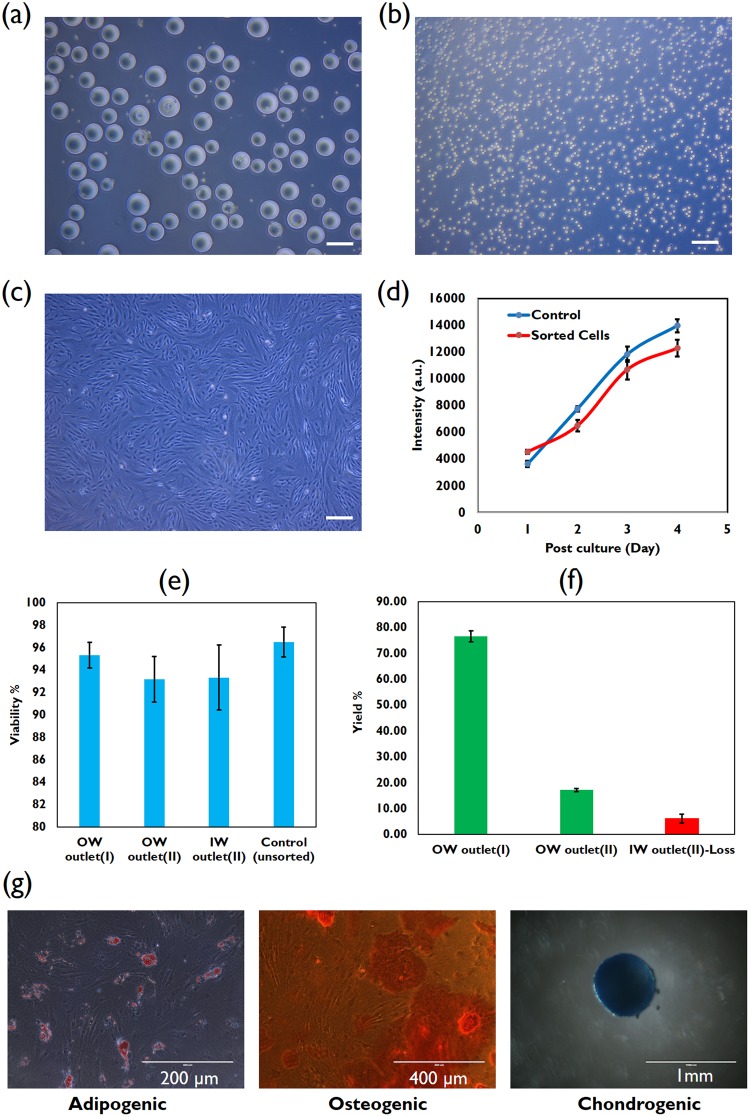
Collected samples from (**a**) the inner wall (IW) outlet and (**b**) the outer wall (OW) outlet (scale bar is 200 µm). (**c**) monolayer culture in a 6-well plate in day 5 and (**d**) PrestoBlue assay displays semi-quantitative growth of harvested BM-hMSCs compared to control sample. (**e**) Viability of harvested BM-hMSCs from the OW outlet at pass (I,II) and the IW outlet at pass (II) compared to initial viability. (**f**) Yield gains from the OW outlet at first and second pass and loss from the IW outlet at second pass. (**g**) Multipotency assay of harvested BM-hMSCs using Oil Red O, Alizarin red and Alcian blue solution to stain lipid droplets (adipocytes, left panel), calcium deposits (osteoblasts, central panel), and extracellular matrix proteins (chondrocytes, right panel) respectively.

Figure 4a,b display collected samples from IW and OW outlet after the second pass, respectively. Clearly, the contrast between the two samples displays cells depleted in the IW outlet (Fig. 4a) and conversely removal of MCs from the OW outlet (Fig. 4b). The purity of harvested cells was able to reach ~99% which is a crucial attribute. Harvested cells were cultured subsequently in a plastic tissue culture, as shown in Fig. 4c, while maintaining their spindle-like morphology^42^. Furthermore, a semi-quantitative assay using PrestoBlue demonstrated that the proliferation of harvested cells *in vitro* compared to a control sample of unsorted cells was similar for 4 days post culture, Fig. 4d. With respect to cell viability, Fig. 4e illustrates the negligible reduction in viability of harvested cells from 96.5 ± 1.32% (control sample, n = 3) to 95.3 ± 1.15% (OW outlet-I) and 93.16 ± 2% (OW outlet-II) after the first and second pass respectively. After counting, cell harvest results showed 76.62 ± 2.1% and 17.21 ± 0.6% cell recovery from the OW outlet at first and second pass respectively, and 6.16 ± 1.80% cell loss through the IW outlet at the end of process displayed in Fig. 4f. The sum of yield (sum of cells harvested from the OW outlet over the total cell harvest from all outlets) can reach ~94%.

Quality of the harvested BM-hMSCs can be checked by a multipotency test to assess whether the cells are capable of differentiating into adipocytes, chondrocytes, and osteoblasts. Results from this multipotency test indicated that the multipotency of the harvested BM-hMSCs was retained and not compromised. After 28 days of trilineage differentiation into the adipogenic, chondrogenic, and osteogenic lineages, the cells showed positive staining for the 3 differentiated lineages, as shown in Fig. 4g. Formation of lipid vacuole visualized by Oil Red O confirmed adipocytic phenotype^41,42^, the presence of stained Alcian Blue-glycosaminoglycan complex showed typical chondrocytic phenotype^41,43^ and mineralized matrix stained by Alizarin Red S indicated osteoblastic phenotype^41,44^.

Close scrutiny of shear rate distribution accomplished numerically across the spiral channel at the working flow rate of ~30 mL/min (Fig. S5) reveals that its magnitude is smaller than the shear rate of 3000 s^−1^ recently verified for volume reduction of hMSC using TFF^15^. M oreover, the present configuration of the scaled-up spiral with a decreasing-Dean mode, where outlets are placed at the outermost of the spiral curve, leads to ease of parallelization in a plane and sizable scaling out of the throughput via multiplexing spiral channels, as recently reported by our group^17,43^. As a result, the designed spiral could potentially be stacked up to deliver on a more relevant industrial scale (~1–5 L/min) in practice. This proposed inertial-based filtration method offers beneficial attributes such as a closed system, scalability, and continuous mode (vs. batch processing) that are appreciated for large-scale cell manufacturing in downstream processing^8,44^.

Though there is recently a paradigm shift towards dissolvable MCs to circumvent separation following cell expansion, the majority of commercially available MCs widely adopted are non-dissolvable^1,36^. On the other hand, emerging thermally-responsive MCs for culturing adherent cell types^45,46^ may eliminate the need for enzymatic treatment of cells, such as exposure to trypsin but the MCs still have to be separated. There will likely be advantages and downsides for each option chosen whether using dissolvable MCs or non-dissolvable MCs. Furthermore, the device demonstrated is versatile, and it can perform MC retention, removal of large-sized particulates from manufactured cell products such as MSCs.

## Conclusion

We have developed a scaled-up trapezoidal spiral channel (at millimeter dimensions) that removes microcarriers from cell suspensions. A series of trapezoidal and rectangular spirals were investigated. All channels possessed minimum height of 0.5 mm, i.e. particle clogging ratio of K ≈ 0.35, since any smaller channel height increased the potential of clogging of the channel. It was found that elevating the average channel height by 0.2 mm, while reducing the average particle clogging ratio (K = a/H) from 0.26 to 0.20, caused MC partial focusing only at a noticeably low Re number of ~56 though there is no theoretical constraint yet reported. A trapezoidal spiral with ultra-low-slope slanted wall (Tan(α) = 0.0375) developed MC focusing near the inner wall at high Re numbers ranging from ~300 to ~400. This contributed to a high-throughput device while maintaining high MC filtration efficiency (E ≈ 99%) as a crucial trait. The present channel was implemented to remove MCs from hMSC suspension at ~30 mL/min (Re~326.5) with total yield ~94% after two passes. Overall cell viability was maintained at 95.3 ± 1.15% (n = 3) and 93.16 ± 2% (n = 3) after first and second pass, respectively. Harvested cells demonstrated all potential trilineage differentiations and morphology as expected.

## Electronic supplementary material

Supplementary Information

VIDEO S1-Rectangular Spiral-14 mL/min

VIDEO S2-Ultra-low-slope Trapezoidal spiral-30ml/min

VIDEO S3-Ultra-low-slope Trapezoidal spiral-30ml/min at Bifurcation

## Data Availability

All the raw data will be made available in response to any reasonable requests, after the publication and completion of necessary intellectual property-related steps.
