# Supplementary material for: Inertial-Based Filtration Method for Removal of Microcarriers from Mesenchymal Stem Cell Suspensions
Source: Sci Rep. 2018 Aug 20;8:12481. doi: 10.1038/s41598-018-31019-y (PMC6102204; doi:10.1038/s41598-018-31019-y)
Supplement: Supplementary file 1 — Supplementary Information [file 41598_2018_31019_MOESM1_ESM.pdf]

Inertial-based filtration method for removal of microcarriers from mesenchymal stem cell suspensions

Reza Moloudi, Steve Oh, Chun Yang, Kim Leng Teo, Alan Tin-Lun Lam, Majid Ebrahimi Warkiani, and May Win Naing

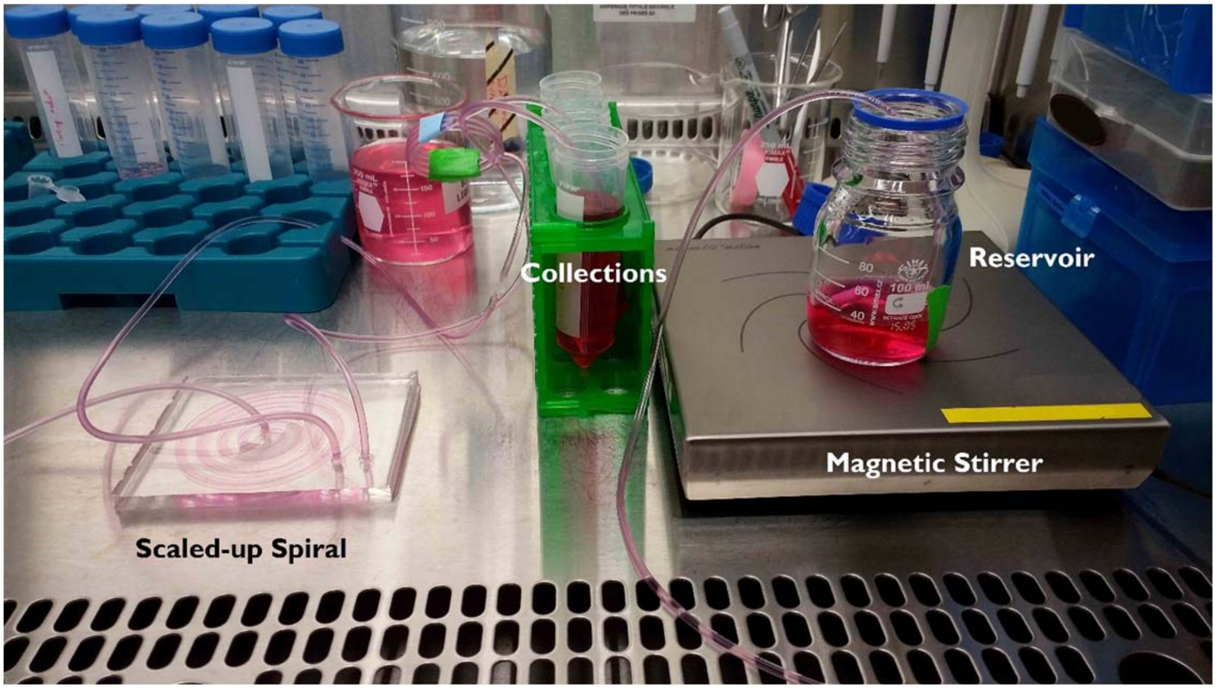

Fig. S1 Experimental set up for separation of microcarriers from hMSCs

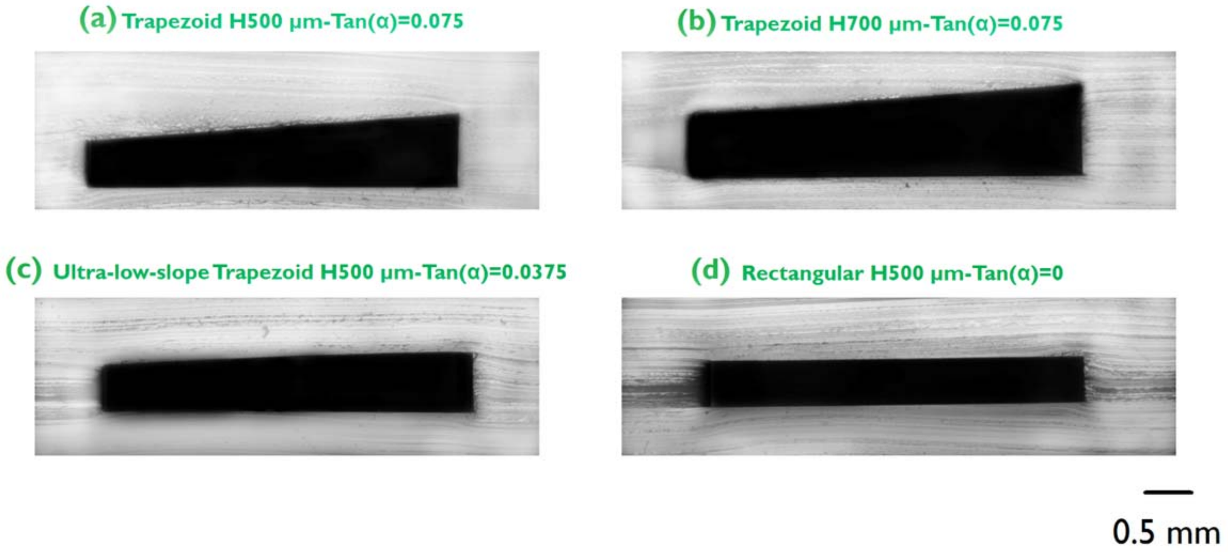

Fig. S2 The cross-sections of fabricated trapezoid and rectangular spiral channels. All channel's widths are fixed at 4 mm.

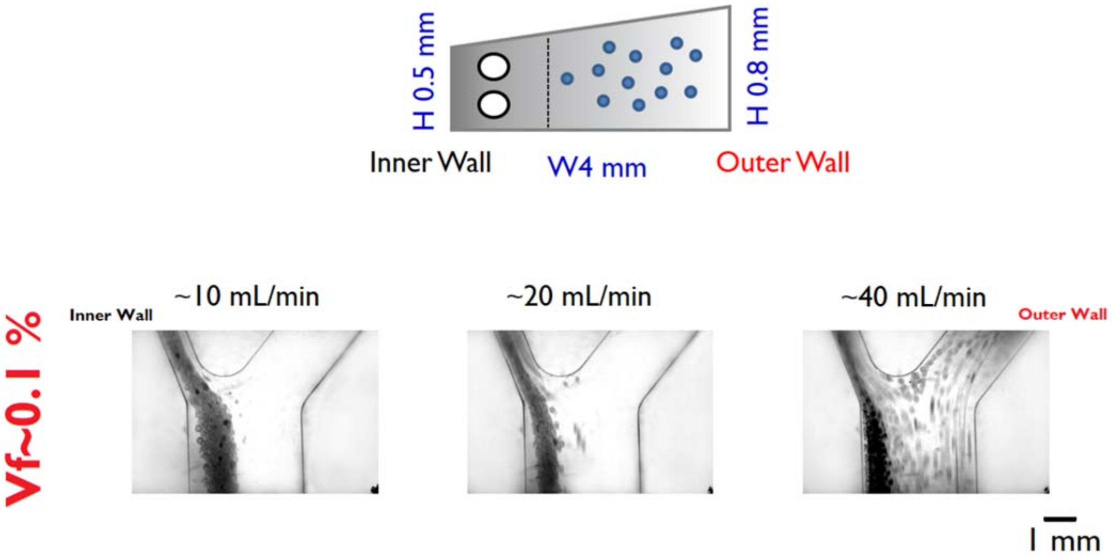

Fig. S3 Microcarrier focusing dynamics at the bifurcation of trapezoid spiral channel with H=500 µm, Tan(α)=0.075 when the MC volume fraction is as low as ~0.1%. It shows good-focusing and separation of MCs from the inner wall outlet at ~20 mL/min.

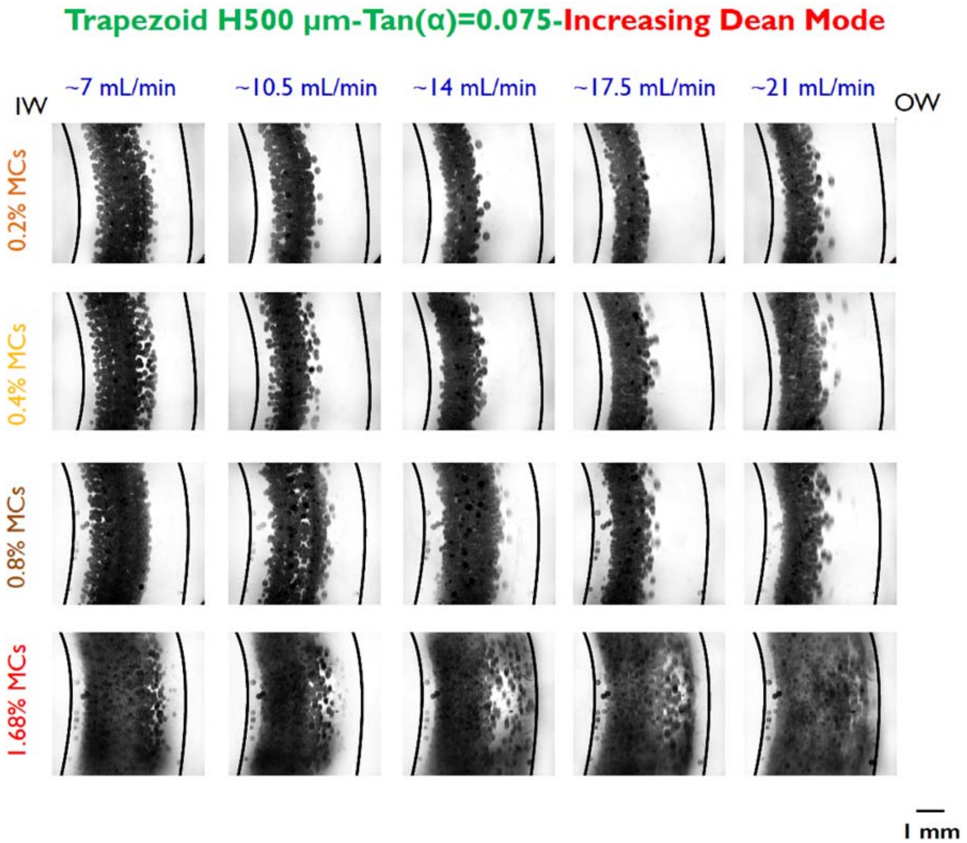

Fig. S4 Microcarrier focusing dynamics in trapezoid spiral channel with H=500 µm, Tan(α)=0.075 when Dean number increases across the spiral channel.

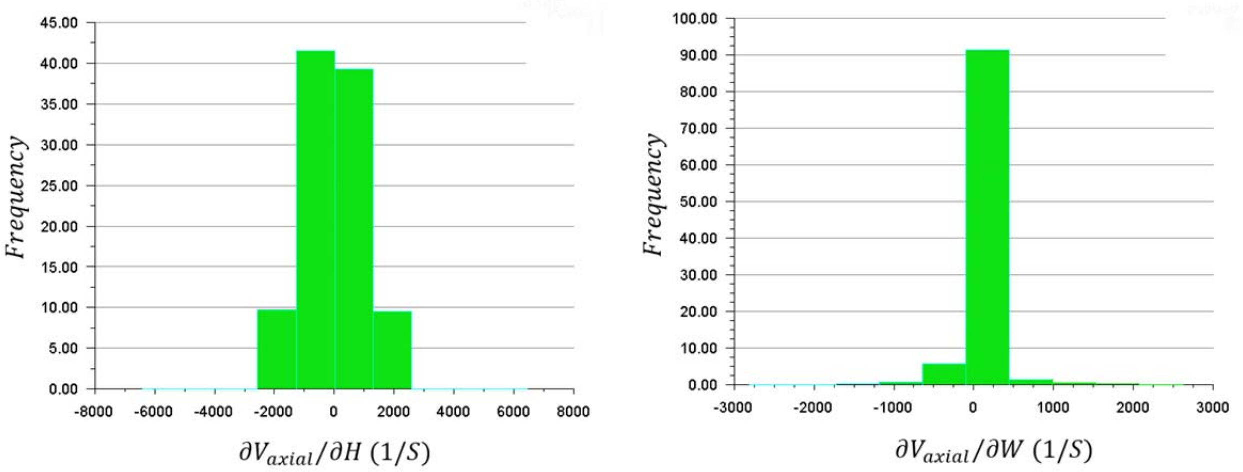

Fig. S5 Histogram of two major shear rates across the ultra-low-slope trapezoid spiral channel, H=500 µm, Tan(α)=0.0375.

Table S1. MC count from the outer wall outlet sample (n=3) using the ultra-low-slope trapezoidal spiral

MC volume fraction %	0.2	0.4	0.8	1.6
C_o (MCs/mL) Outer wall outlet	0	7.6±2.5	24.3±4	41.6±7.6
C_s(MCs/mL) Reservoir	~750	~1500	~3000	~6000
E %	100	99.4	99.19	99.3

Note: To perform microcarrier (MC) count of the outer wall outlet collection, due to low concentration, 1-mL sample was dispersed into a petri dish and subsequently MCs were counted manually on the microscope stage using bright field.
